# Assessing infant sleep practices and other risk factors of SIDS in Zambia: a cross-sectional survey of mothers in Lusaka, Zambia

**DOI:** 10.1186/s12887-022-03712-5

**Published:** 2022-11-15

**Authors:** Godwin K. Osei-Poku, Lawrence Mwananyanda, Patricia A. Elliot, William B. MacLeod, Somwe Wa Somwe, Rachel C. Pieciak, Christopher J. Gill

**Affiliations:** 1 Department of Global Health, Boston University School of Public Health, 801 Massachusetts Avenue, Crosstown Center, 3rd Floor, Boston, MA 02118 USA; 2 Department of Community Health, Boston University School of Public Health, 801 Massachusetts Avenue, Crosstown Center, 4th Floor, Boston, MA 02118 USA; 3 Department of Pediatrics, School of Medicine, University of Zambia, Lusaka, Zambia

**Keywords:** SIDS, SUID, Bedsharing, Sleep position

## Abstract

**Background:**

Having infants sleep with their parents and sleeping face down or on their sides are the two most proximate and modifiable risk factors for sudden infant death syndrome (SIDS). Little is known about the burden of SIDS or the prevalence of these risk factors in Africa. Our primary objective was to determine the prevalence of modifiable risk factors of SIDS in Lusaka, Zambia.

**Methods:**

We conducted cross-sectional surveys with recent mothers of infants aged < 1 year across two busy urban clinic sites in Lusaka, Zambia. We used log-binomial regression analysis to identify factors predictive of bedsharing and prone sleeping.

**Results:**

Surveys were conducted with 478 mothers between April-May 2021. The sleep-related risk factors, bedsharing and side sleeping, were widely prevalent. 89.5% of respondents indicated that they share a bed with the infant during sleep, 73.0% preferred putting their baby on its side, and 19.9% preferred the prone position. Only 6.7% of respondents described using the safer, supine position. Age of infant was the only factor which was predictive of prone sleeping. Infants > 2 months old were twice as likely to be put to sleep in a prone position compared to infants aged less than 2 months old. Mothers reported that they rarely (24.1%) received advice from medical caregivers to use the supine position. Maternal use of alcohol (12.0%) and tobacco (0.8%) during pregnancy were uncommon.

**Conclusions:**

Bedsharing and placing the infant to sleep on the side were commonly reported among the mothers we interviewed. Whether this represents an opportunity to reduce SIDS in Zambia is unclear since accurate data on the burden of SIDS in Zambia is not available. There is a need for increased awareness of SIDS and more prospective data collection on its burden and related risk factors in these African populations.

## Background

The most significant risk factors of sudden infant death syndrome (SIDS) are related to the infant’s sleeping environment and position. Previous research from high-income countries suggests a more than 2 fold increased risk of SIDS with prone sleeping and bed-sharing [1, 2]. Over the years, public health campaigns have made critical strides in reducing infant mortality from SIDS by targeting major risk factors such as prone sleeping. These initiatives, including the “back to sleep” campaign in the U.S., have accounted for the more than 50% reduction in SIDS-related infant mortalities [3]. Based on this, the American Academy of Pediatrics recommends that infants should be placed to sleep on the back and on a separate sleep surface, ideally in the same room as the mother [4].

It is informative that the risk factors for SIDS and other sleep-related infant deaths, such as accidental suffocation or strangulation in bed (ASSB), are very similar and hence difficult to parse based on clinical history [5]. Other risk factors commonly associated with an increased risk of SIDS include little or no prenatal care, maternal age less than 20 years, prematurity or low birth weight, and maternal smoking [6, 7]. There is also a strong association between SIDS and poverty, evidenced by its link with low maternal education, single marital status and being black [8, 9]. In addition, there is a strong association between SIDS and maternal substance use, including alcohol, and cannabis use, which increase the risk of the child being smothered accidentally by an intoxicated parent. Air pollution from indoor household cooking is a more recently identified risk factor for SIDS [10].

To date, only a handful of studies, mostly from South Africa, have assessed the prevalence of SIDS risk factors in Africa [8, 11–14]. In Zambia specifically, where our team has worked for the past 20 years, little is known regarding the prevalence of these risk factors. To fill this knowledge gap and inform public health policy in Zambia, we conducted cross-sectional surveys with mothers of infants aged 1 year or less in Lusaka to explore the risk factors of SIDS and other sleep-related infant deaths. Surveys were administered over 4 weeks between April and May 2021. To our knowledge, this was the first study to assess the risk factors of SIDS in Zambia.

### Study objective

The primary objective of this study was to determine the prevalence of modifiable risk factors for SIDS and other sleep-related infant deaths in Lusaka, Zambia. Our secondary objective was to determine if there are any significant differences in the prevalence of these risk factors between two peri-urban townships with different socio-economic profiles in Lusaka, Zambia.

## Methods

### Study location/sites

Data collection occurred at postnatal clinics across two government-run level one hospitals in Lusaka: the Chilenje and Chawama hospitals [15]. These sites were selected for several reasons. First, both sites are in large, urban communities in Lusaka with a high burden of infant mortality. Second, they are demographically distinct from each other. Chilenje attends to a wealthier and more advantaged population in Lusaka, whereas the Chawama township is one of the most densely populated and economically stressed parts of the city, with very high rates of poverty. Third, and for the same reason, both are sites where the study team has invested time and resources to develop relationships with local staff that facilitate working in these communities.

Both hospitals offer in-patient and out-patient services with maternal and child health departments that offer daily antenatal, labor and delivery, postnatal, family planning and child health services [16]. Chilenje hospital is a 46-bed hospital serving a population of ~ 95,000 [15]. Chawama is a 30-bed hospital serving a population of ~ 129,000 [15].

### Recruitment and sampling

Surveys targeted mother-baby pairs at each study site. Through a non-probability sampling method, designated nurses at each study site identified mothers who met the inclusion criteria for participation in the study. Eligible participants were aged 18 to 49 years, nursing an infant aged under 1 year of age, resident within the catchment area of each study hospital and able to give either verbal or written consent for participation in the study.

### Data collection

A pretested 64-item questionnaire was administered to each study participant at the clinic on mobile devices through an online survey software, Qualtrics (Qualtrics XM Inc., Seattle, WA). The questionnaire was adapted from the U. S Center for Disease Control and Prevention’s sudden unexpected infant death investigation reporting form (SUIDIRF), [17] and the 2018 demographic and health survey (DHS) of Zambia [18]. Both are publicly available validated data collection instruments. Additional questions were developed by the study team in consultation with a Zambian pediatrician, Dr. Somwe Wa Somwe.

Questionnaires were administered on the same day respondents gave consent to reduce non-response. The questionnaire collected information on maternal knowledge and awareness of SIDS, previous family history of sudden infant death, infant sleeping practices, maternal use of substances (alcohol, tobacco, or cannabis) during pregnancy, antenatal care, and breastfeeding practices.

### Variables

Through our study, we aimed to measure the prevalence of the sleep-related risk factors among the Zambian mothers. Our primary outcome of interest was bedsharing which was defined as routinely sharing a bed with the mother, father, or other relatives (sibling, aunt, grand parent, etc.). We classified infant sleeping positions into three categories: prone (tummy/stomach), supine (sleeping on the back), or lateral (sleeping on the side). Information on the routine sleeping place for the infant was also noted. From each mother, we also collected demographic information including age, highest educational attainment, occupation, parity, marital status. We also collected demographic features of the infant including sex, age, and presence of birth defects. We assessed maternal use of alcohol, and tobacco during pregnancy by asking mothers to indicate if they smoked cigarettes or used alcohol during their most recent pregnancy. Survey responses were checked daily for completeness.

### Sample size determination

Our sample size requirements for this survey were driven by our primary outcome of interest, bed-sharing. A literature search did not yield local prevalence rates of bed-sharing in Zambia. However, a qualitative report by the American Institute of Research (AIR) and UNICEF in 2018 [19] revealed that the majority of mothers in rural Zambia indicated they sleep with their baby in the same bed until the babies are at least 2 years old. In addition, a study in South-Eastern Nigeria reported a 67% prevalence rate of infant bed-sharing with adults [8]. Thus, we assumed a 50% prevalence rate of bed-sharing in Zambia and estimated our sample size using the formula: Z^2^ P (1-P)/d^2^ where Z is the statistic corresponding to the level of confidence (Z = 1.96 for 95% Confidence Interval), P is the expected prevalence of the outcome and d is the level of precision. Using a 95% confidence level and 5% level of precision, the required sample size was 384 mother-baby dyads. To account for a 20% non-consent rate, we estimated that we would need to enrol up to 480 participants (240 from each study site).

### Statistical analysis

All statistical analyses were conducted in SAS 9.4. Frequencies/proportions, means and standard deviations were calculated as appropriate. Differences in proportions of categorical variables were tested for statistical significance using chi-square test. Differences in mean of continuous variables were tested for significance using a t-test. We used log-binomial regression to calculate associations with demographic factors predictive of bed-sharing and the prone sleeping position. We report prevalence ratios (PR) and 95% confidence intervals (95% CI) as our measures of effect. All statistical analyses were conducted at a 0.05 significance level.

## Results

### Demographic features

We consented and enrolled 478/498 (97%) of the mothers that were approached. Table 1 summarizes the demographic features of mothers and their infants, stratified by residence in Chawama or Chilenje compounds. On average, mothers were 28 years old (*std* = 6.1 years). Most were married or cohabiting, 80.5% (385/478), and had 1-2 children 60.0% (287/478). Roughly half were unemployed or had completed secondary school. There were several notable differences between survey respondents at Chawama compared to Chilenje. Survey respondents at Chawama were slightly younger and were far less likely to have attended post-secondary school (9.6% vs 52.9%). There were more single/divorced/widowed mothers at Chilenje compared to Chawama (26.1% vs 12.9%). In addition, more than half of the mothers in Chilenje were formally employed (57.1%) compared to only a little over a third of mothers in Chawama (37.5%).

**Table 1:** Population characteristics of survey respondents

Characteristic	Total *N =* 478	Study Site		
		Chawama ***N =*** 240	Chilenje ***N =*** 238	***P***-value†
Maternal Characteristics				
*Age, mean (std.)*	28.0 (6.1)	26.7 (5.5)	29.4 (6.5)	<.0001‡
*Age in years, n (%)*				<.0001
< 20	31 (6.5%)	16 (6.7%)	15 (6.3%)	
20-29	272 (56.9%)	160 (66.7%)	112 (47.1%)	
30-39	153 (32.0%)	59 (24.6%)	94 (39.5%)	
> =40	22 (4.6%)	5 (2.1%)	17 (7.1%)	
*Marital status, n (%)*				0.0003
Single/Divorced/Widowed	93 (19.5%)	31 (12.9%)	62 (26.1%)	
Married/Co-habiting	385 (80.5%)	209 (87.1%)	176 (73.9%)	
*Highest Education*^a^*, n (%)*				<.0001
Never attended school	6 (1.3%)	5 (2.1%)	1 (0.4%)	
Primary	82 (17.2%)	64 (26.7%)	18 (7.6%)	
Secondary	240 (50.2%)	148 (61.7%)	92 (38.7%)	
Postsecondary	149 (31.2%)	23 (9.6%)	126 (52.9%)	
*Occupation*^a^*, n (%)*				<.0001
Salaried employee/Self employed	226 (47.3%)	90 (37.5%)	136 (57.1%)	
Unemployed	251 (52.5%)	149 (62.1%)	102 (42.9%)	
*Parity*				0.9850
1-2	287 (60.0%)	144 (60.0%)	143 (60.1%)	
> =3	191 (40.0%)	96 (40.0%)	95 (39.9%)	
Infant Characteristics				
*Age in months, n (%)*				
< 2mo	93 (19.5%)	65 (27.1%)	28 (11.8%)	<.0001
2-4mo	159 (33.3%)	97 (40.4%)	62 (26.1%)	
5-7mo	98 (20.5%)	32 (13.3%)	66 (27.7%)	
8-10mo	78 (16.3%)	29 (12.1%)	49 (20.6%)	
11mo	50 (10.5%)	17 (7.1%)	33 (13.9%)	
*Gender/Sex, n (%)*				
Male	249 (52.1%)	125 (52.1%)	124 (52.1%)	0.9969
Female	229 (47.9%)	115 (47.9%)	114 (47.9%)	

^**a**^Maternal education and occupation were unknown for one participant; † *p-*value from chi-square test; ‡ *p-*value from t-test

### Awareness of SIDS

A large proportion, 67.6% (323/478) of survey respondents, reported that they knew someone or had heard of someone losing an infant suddenly and unexpectedly during sleep, indicating a high level of awareness of SIDS. A significantly larger proportion of respondents indicated that they were familiar with SIDS in Chawama compared to respondents in Chilenje (72.9% vs 62.2%) (PR: 1.16, 95% CI: 1.03, 1.32) (Data not shown).

### Risk factors intrinsic to the infant

Overall, 7.3% (24/331) of respondents reported previously losing an apparently healthy infant to suspected SIDS. The reported rate of suspected SIDS appeared higher among twins. Overall, 12% (3/25) of mothers with twins reported losing one twin suddenly and unexpectedly during sleep. Birth defects were not common in this population, or at least were not commonly identified by the mothers. Only 1.3% (6/478) of mothers reported a history of apparent birth defects in their infant. We assessed prematurity by asking mothers to estimate if the infant was born early compared to their expected delivery date. Overall, 19% (91/478) of mothers reported giving birth to the infant early. Related to this, only 9.8% (47/478) of infants were reported to have been born with a birth weight under 2500 g (low birth weight). The risk of a previous history of suspected SIDS was not significantly different between mothers who reported giving birth to the infant early (PR: 0.66, 95% CI: 0.20, 2.14) or with low birth weight compared to those who did not (PR: 1.74, 95% CI: 0.63, 4.80). Note that the lack of an association between pre-term births and suspected SIDS should be interpreted cautiously, given that gestational age estimates are notoriously inaccurate in the absence of ultrasound dating.

Table 2 summarizes the prevalence of risk factors intrinsic to the infant by study site. Participants in Chawama were more likely to report higher rates of these intrinsic risk factors compared to participants in Chilenje. While 8.5% (17/176) of mothers at Chawama reported a previous history of suspected SIDS, only 5.8% (9/155) of mothers at Chilenje reported such losses (PR: 1.47, 95% CI: 0.66, 3.26). In addition, slightly more infants were low birthweight in Chawama (10.8%) compared to Chilenje (8.8%). Similarly, more infants were reported as premature (born “early”) at Chawama (24.2%) compared to Chilenje (13.9%) (PR:1.74, 95% CI: 1.18, 2.57).

**Table 2:** Prevalence of risk factors intrinsic to the infant

Characteristic	Total		Study Site		
	n/N	***Prevalence % (95% CI)***	Chawama n/N	Chilenje n/N	***PR (95% CI)***
*Previous history of suspected SIDS*^a^	24/331	7.3 (4.7, 10.6)	15/176	9/155	1.47 (0.66, 3.26)
*Twin birth*	25/478	5.2 (3.4, 7.7)	13/240	12/238	1.07 (0.50, 2.30)
*Previous suspected SIDS in twins*	3/25	12.0 (2.6, 31.2)	2/13	1/12	1.85 (0.19, 17.84)
*Birth defects*	6/478	1.3 (0.5, 2.7)	4/240	2/238	1.98 (0.37, 10.68)
*Birth weight*					
< 2500 g	47/478	9.8 (7.4, 12.9)	26/240	21/238	1.24 (0.72, 2.14)
> =2500	429/478	89.7 (84.1, 90.2)	212/240	217/238	0.98 (0.92, 1.04)
*Time of delivery*					
Early	91/478	19.0 (15.6, 22.9)	58/240	33/238	1.74 (1.18, 2.57)
On time/late	387/478	80.9 (77.2, 84.4)	182/240	205/238	0.88 (0.81, 0.96)

^**a**^This was asked of only mothers with more than 1 previous live birth (parity > 1); The rate of suspected SIDS should be interpreted cautiously since this is based on self-report; we could not confirm these rates objectively with medical records or autopsy

### Infant sleep practices

We examined the risk of SIDS due to infant sleep practices at both Chawama and Chilenje. Co-sleeping with an infant either on the same bed or in the same room was universally practised. Nearly all mothers (89.5%) reported that their infant shared a sleep surface with an adult or other person. Conversely, very few mothers (5.6%) reported that the infant slept alone in a crib. There was no statistically significant difference in the prevalence of bedsharing between participants in Chawama and Chilenje (PR, 0.99, 95% CI: 0.93, 1.05). When asked how often they share a bed with the baby, most participants, 47% (224/478) reported “always” sleeping with the infant.

Table 3 summarizes infant sleep positions and bedding types across the two cohorts. Overall, the practice of putting infants to sleep in the lateral position was clearly preferred over having infants sleep supine or prone. Most participants (73.0%) reported placing their infant to sleep in the lateral position, while only 6.7% reported putting babies to sleep on their backs. When asked about the best position and location to place an infant to sleep, the majority of respondents indicated that sleeping in the same bed with the mother (60.5%, 289/478) and being placed in the lateral position (70.1%, 335/478) were best. Only 24.1% (115/478) of mothers indicated that they had ever been advised by medical personnel to put the infant to sleep on the back; however, most respondents (65.5%) indicated a willingness to change their sleep practices if their own practices were not aligned with medically guided information about safe infant sleep practices.

**Table 3:** Prevalence of risk factors related to infant sleep practices

Characteristics	Total		Study Site		
	n/N	***Prevalence %*** ***(95% CI)***	Chawama n/N	Chilenje n/N	***PR (95% CI)***
*Infant’s usual sleeping place*					
Parents bed	445/478	93.1 (90.4, 95.2)	229/240	216/238	1.05 (1.00, 1.10)
Crib or Bassinet	27/478	5.6 (3.8, 8.1)	7/240	20/238	0.35 (0.15, 0.81)
Sofa or Floor	6/478	1.2 (0.5, 2.7)	4/240	2/238	1.98 (0.37, 10.73)
*Infant sleeps alone in a separate room*	19/478	4.0 (2.4, 6.1)	8/240	11/238	0.72 (0.29, 1.76)
*Bedsharing with infant*	428/478	89.5 (86.4, 92.1)	214/240	214/238	0.99 (0.93, 1.05)
*Position placed to sleep*^a^					
Lateral	349/478	73.0 (68.8, 76.9)	192/240	157/238	1.21 (1.09, 1.36)
Supine	32/478	6.7 (4.6, 9.3)	9/240	23/238	0.39 (0.18, 0.82)
Prone	95/478	19.9 (16.4, 23.7)	38/240	57/238	0.66 (0.46, 0.96)
*Type of Mattress*					
New	143/478	29.9 (25.8, 34.2)	55/240	88/238	0.62 (0.47, 0.83)
Used	328/478	68.6 (64.3, 72.8)	181/240	147/238	1.23 (1.09, 1.38)
*Sleep surface*					
Soft	199/478	41.6 (37.2, 46.2)	108/240	91/238	1.18 (0.95, 1.46)
Medium	127/478	26.6 (22.7, 30.8)	69/240	58/238	1.18 (0.87, 1.59)
Firm/Hard	118/478	24.7 (20.9, 28.8)	52/240	66/238	0.78 (0.57, 1.07)
*Best position to place infant*					
Lateral	335/478	70.1 (65.8, 74.2)	180/240	155/238	1.15 (1.02, 1.30)
Supine	38/478	7.9 (5.7, 10.7)	11/240	27/238	0.40 (0.21, 0.80)
Prone	98/478	20.5 (17.0, 24.4)	45/240	53/238	0.84 (0.59, 1.20)
*Best place for infant to sleep*					
In bed with mother	289/478	60.5 (55.9, 64.9)	145/240	144/238	1.00 (0.86, 1.15)
Same room with mother	97/478	20.3 (16.8, 24.2)	38/240	59/238	0.64 (0.44, 0.92)
Alone in a crib	82/478	17.2 (13.9, 20.8)	53/240	29/238	1.81 (1.20, 2.75)
In separate room	6/478	1.3 (0.5, 2.7)	2/240	4/238	0.50 (0.09, 2.68)
*Advise by medical personnel to lay baby on back*	115/478	24.1 (20.3, 28.2)	59/240	56 (238)	1.04 (0.76, 1.44)
*Willingness to change infant sleep practices*	313/478	65.5 (61.0, 69.7)	151/240	162/238	0.92 (0.81, 1.05)

^**a**^ Mothers were asked to indicate the usual position in which the infant was placed to sleep

By study site, significantly more mothers at Chawama (80.0%, 192/240) placed their baby to sleep in the lateral position compared to Chilenje 66.0% (157/238) (PR: 1.21, 95% CI: 1.09, 1.36)). Interestingly, the prone and supine positions were less frequently used by mothers at Chawama compared to mothers at Chilenje; (PR: 0.66, 95% CI: 0.46, 0.96) and (PR: 0.39, 95% CI: 0.18, 0.82) respectively. The type of mattress babies slept on was also reported to be mostly used (68.6%, 328/478) and soft (41.6%, 199/478).

### Multivariate analysis of factors predictive of bedsharing and prone sleeping

In multivariate analyses, none of the maternal or infant demographic factors was significantly associated with bedsharing (Table 4). However, infant age was significantly associated with prone sleeping, with older infants (between 2 and 10 months) more likely to be placed to sleep in the prone position. For example, infants aged 2-4 months had a 2.33 increased risk of prone sleeping compared to infants aged less than 2 months (PR: 2.33, 95% CI: 1.16, 4.71).

**Table 4:** Multivariate analysis of factors predictive of bedsharing and prone sleeping

Characteristic	Bedsharing *PR (95% CI)*	Prone sleeping *PR (95% CI)*
*Maternal age in years, n, %*		
20-29	*1.00*	*1.00*
< 20	1.06 (0.93, 1.21)	1.47 (0.75, 2.88)
30-39	1.02 (0.92, 1.12)	0.89 (0.57, 1.39)
> =40	0.97 (0.80, 1.17)	1.02 (0.44, 2.37)
*Marital status, n (%)*		
Married/Co-habiting	*1.00*	*1.00*
Single/Divorced/Widowed	0.92 (0.83, 1.03)	1.16 (0.74, 1.81)
*Maternal Education, n (%)*		
Primary or less	0.99 (0.88, 1.13)	1.02 (0.54, 1.91)
Secondary	0.97 (0.86, 1.10)	1.04 (0.66, 1.62)
Postsecondary	*1.00*	*1.00*
*Maternal Occupation, n (%)*		
Salaried or Self employed	*1.00*	*1.00*
Unemployed	1.02 (0.93, 1.11)	0.95 (0.63, 1.43)
*Infants age in months, n (%)*		
0-1mo	*1.00*	*1.00*
2-4mo	0.96 (0.87, 1.07)	2.33 (1.16, 4.71)^a^
5-7mo	0.96 (0.84, 1.09)	2.25 (1.08, 4.68)^a^
8-10mo	1.05 (0.93, 1.19)	2.44 (1.16, 5.13)^a^
11-12mo	0.99 (0.85, 1.15)	1.63 (0.66, 4.03)
*Infant’s gender/Sex, n (%)*		
Female	*1.00*	*1.00*
Male	1.03 (0.95, 1.12)	0.97 (0.67, 1.40)
*Study Site*		
Chilenje	*1.00*	*1.00*
Chawama	0.99 (0.88, 1.12)	0.75 (0.48, 1.17)

^a^Significant at alpha< 0.05; Models adjusted for maternal age, marital status, education and occupation, infant age, gender/sex, and study site

### Maternal use of substances and adequacy of antenatal care and breastfeeding

We examined the relationship between maternal substance use during pregnancy at both sites (Table 5). Some mothers (12.1%) reported alcohol use in their most recent pregnancy. By study site, mothers at Chawama were less likely to report the use of alcohol in their most recent pregnancy compared to mothers at Chilenje (PR: 0.61, 95% CI: 0.37, 0.99). Very few mothers (0.8%) reported any cigarette use during their most recent pregnancy. Similarly, the use of other tobacco products during pregnancy was also low (1.0%, 5/478). Despite low reports of cigarette and other tobacco use in pregnancy, mothers often reported infant exposure to secondhand smoke. Among the mothers surveyed, 18.6% reported that they had an exposure to cigarette smoke during their most recent pregnancy, and 17.6% reported that their infant had been exposed to cigarette smoke in the months after birth respectively. No mother reported any use of marijuana. We note that recreational/medicinal use of marijuana is illegal in Zambia, and this could have influenced their willingness to respond openly.

**Table 5:** Maternal use of alcohol, and tobacco, and adequacy of antenatal care and breastfeeding

Characteristic	Total		Study Site		
	n/N	***Prevalence % (95% CI)***	Chawama n/N	Chilenje n/N	***PR (95% CI)***
*Prenatal alcohol use*	58/478	12.1 (9.3, 15.4)	22/240	36/238	0.61 (0.37, 0.99)
*Prenatal smoking*	4/478	0.8 (0.2, 2.1)	1/240	3/238	0.33 (0.03, 3.14)
*Secondhand smoke exposure*					
Prenatal	89/478	18.6 (15.2, 22.4)	51/240	38/238	1.31 (0.90, 1.92)
Postnatal	84/478	17.6 (14.3, 21.3)	45/240	39/238	1.14 (0.78, 1.69)
*Prenatal OTP (nsunko)*	5/478	1.0 (0.3, 2.4)	2/240	3/238	0.65 (0.11, 3.90)
*Attended antenatal care*	477/478	99.8 (98.8, 100.0)	240/240	237/238	1.00 (1.00, 1.01)
*Start of antenatal care*					
1st Trimester	173/478	36.2 (31.9, 40.7)	73/240	100/238	0.72 (0.57, 0.92)
2nd Trimester	253/478	52.9 (48.3, 57.5)	140/240	113/238	1.23 (1.04, 1.46)
3rd Trimester	46/478	9.6 (7.1, 12.6)	24/240	22/238	1.08 (0.62, 1.88)
*Antenatal care visits*					
1-2	38/478	7.9 (5.7, 10.8)	21/240	17/238	1.23 (0.66, 2.26)
3-4	172/478	36.0 (31.7, 40.5)	111/240	61/238	1.80 (1.40, 2.33)
5-6	175/478	36.6 (32.3, 41.1)	86/240	89/238	0.96 (0.76, 1.21)
> 7	85/478	17.8 (14.5, 21.5)	17/240	68/238	0.25 (0.15, 0.41)
*General infant feed*					
Breastmilk only	271/478	56.7 (52.1, 61.2)	172/240	99/238	1.72 (1.45, 2.04)
Breastmilk and Complementary	191/478	40.0 (35.5, 44.5)	65/240	126/238	0.51 (0.40, 0.65)
Not BF	16/478	3.3 (1.9, 5.4)	3/240	13/238	0.23 (0.07, 0.79)
*Exclusive breastfeeding* ^a^					
< 3mo	29/205	14.1 (9.7, 19.7)	5/67	24/138	0.43 (0.17, 1.08)
3-4mo	45/205	22.0 (16.5, 28.3)	18/67	27/138	1.37 (0.82, 2.31)
5-6mo	120/205	58.5 (51.5, 65.4)	37/67	83/138	0.92 (0.71, 1.19)
7+ mo	11/205	5.4 (2.7, 9.4)	7/67	4/138	3.60 (1.09, 11.9)

Abbreviations: *OTP* Other Tobacco Products, *BF* Breastfeeding

^**a**^ This was limited to infants on complementary feeding

Attendance at antenatal care was nearly universal in this population (99.8%, 477/478). Most participants started antenatal care in the 2nd trimester of pregnancy (52.9%, 253/478), with the majority completing 5–6 visits (36.6%, 175/478). Survey respondents also reported high levels of breastfeeding; only 3.3% (16/478) were not currently breastfeeding. Nearly 57% (271/478) were exclusively breastfeeding at the time of the survey, while 40% (191/478) had introduced complementary feeds in addition to breast milk. Among those who had introduced complementary feed, the majority (58.5%, 120/205) exclusively breastfed for 5–6 months **(**Table 5**)**.

## Discussion

Responses to our survey suggest that sleep-related infant deaths are well-recognized in these Zambian communities. Most mothers (67.6%) indicated they were aware of events of suspected SIDS in their communities, and 7.3% of mothers in this study reported that they personally had lost an infant to suspected SIDS. This rate should however be interpreted cautiously since this is based on self-report; we could not confirm these reported infant losses objectively with medical records or autopsy. Sleep practices that are known to increase the risk of SIDS were commonly reported by the mothers we surveyed. Bedsharing was widely practised, with most mothers indicating that they preferred to share the same bed with the infant. Contrary to recommendations by the American Academy of Pediatrics, very few infants were placed to sleep in the recommended supine position. Instead, mothers we surveyed indicated a preference for placing infants to sleep in the lateral position. In addition, nearly 2 in 10 mothers placed their infant in the prone position to sleep, falling short of the post “back to sleep” target of less than 10% [20]. Significantly, infants aged 2-4 months had an increased risk of being placed prone to sleep, placing the most vulnerable group at an increased risk for SIDS. Moreover, the majority of infants in this study slept on a soft mattress despite evidence to suggest a strong association between sleeping on a soft surface and an increased risk of SIDS [21].

Our findings on these unsafe sleep practices are consistent with that of prior studies in Africa. For instance, the bedsharing rate in our study is not very different from what has been found in the few studies conducted elsewhere in Africa [12, 13] but significantly higher than the rates reported from high-income countries such as the U.S [22]. A key point to note is that these unsafe sleep practices cut across different communities in Lusaka since we found no significant differences in these sleep practices between our two study sites. Given that Zambia is a low-resource country, this finding is not surprising since the socio-economic gradient is not clearly defined between communities. Responses in this study also suggest that mothers are generally willing to change their sleep practices, unfortunately, education from healthcare workers about safe sleep practices is lacking. Nearly a fourth of mothers reported being told by medical personnel to place their infant to sleep on the back.

Maternal substance use did not appear to be highly prevalent in our study. We found an overall prenatal alcohol use rate of 12.1% and a prenatal smoking rate of 0.8%, which are much lower than estimates from South Africa [14, 23]. However, secondhand smoke (SHS) exposure was reported at slightly higher rates in this study. Almost 20% of respondents reported prenatal or postnatal exposure to secondhand smoke. This rate was higher than estimates from Nigeria [12] but lower than what has been reported from South Africa [14, 23]. Both prenatal and postnatal secondhand smoke exposure are recognized as independent risk factors of SIDS [24]. Indeed, the risk of SIDS is higher in babies who are exposed to secondhand smoke after birth than those who are not [25]. Public health campaigns in Zambia should thus focus on reducing prenatal and postnatal secondhand smoke exposure.

Conversely, antenatal care attendance and breastfeeding are potential protective factors against SIDS. Almost all respondents (99.0%) had attended antenatal care, and most completed the recommended 4-6 visits. Breastfeeding was also age-appropriate with almost all infants aged between 1 and 4 months being exclusively breast fed. Additionally, many survey respondents reported  having exclusively breastfed their infant for at least 5-6 months. Several epidemiologic studies indicate that breastfeeding, particularly exclusive breastfeeding, may be protective against the risk of SIDS [26, 27]. The high antenatal care attendance and breastfeeding rates are strengths of Zambia’s public health care delivery system that could be leveraged in a SIDS prevention program.

### Strengths and limitations

The key strength of our analysis is that our study, to the best of our knowledge, is the first to assess the prevalence of known risk factors of SIDS in Zambia. Given how little is known about SIDS in Africa generally, this is a significant contribution to the literature. Our study also had several limitations. First, our findings come from one city in one country at one point in time and may not be generalizable. Our study focused on peri-urban townships in Lusaka and so offer little insight into the prevalence of these factors in rural settings. Responses in this study were self-reported, and some of the concepts addressed could not be quantified precisely. For example, mothers reported whether the infant was born “early”, “on time” or “late”, but these terms are not defined precisely. To address imprecise recall, we asked respondents to focus on their most recent pregnancy and sleep practices for their youngest child. We were also unable to assess some of the genetic and infectious risk factors associated with SIDS. All of this points to the need for further research capable of providing a more definitive estimate of the burden of SIDS and the prevalence of such risk factors among mothers who have lost a child to possible SIDS.

## Conclusions

Prior research has shown that infant sleep position and bedsharing are the two most important predictors of SIDS. Our data confirm that these risk factors are highly prevalent in the population surveyed. Since the risk factors related to sleep are modifiable, this creates an opportunity to intervene and the potential to focus on what appears to be an important, albeit overlooked, potential contributor to infant mortality. While our study provides a snapshot of these risk factors, future research should systematically collect information on these risk factors and determine which of these risk factors are most associated with SIDS in Zambia. In addition, future qualitative studies should focus on understanding the conditions and reasons for the practice of bedsharing and the other unsafe sleep practices we found in this study. Moreover, increased engagement with key stakeholders, including policymakers and community leaders, may be necessary to identify interventions that will be culturally acceptable and feasible to prevent or minimize any unintended consequences of any safe sleep recommendations.

## Data Availability

The datasets used and/or analyzed during the current study are available from the corresponding author on reasonable request.

## Abbreviations

SIDS: Sudden Infant Death Syndrome
SUID: Sudden Unexpected Infant Death
ASSB: Accidental Suffocation and Strangulation in Bed
US: United States
SUIDIRF: Sudden Unexpected Infant Death Investigation Reporting Form
DHS: Demographic and Health Survey
AIR: American Institute of Research
UNICEF: United Nations International Children’s Emergency Fund
